# Supplementary material for: A robust multivariate structure of interindividual covariation between psychosocial characteristics and arousal responses to visual narratives
Source: PLoS One. 2022 Feb 16;17(2):e0263817. doi: 10.1371/journal.pone.0263817 (PMC8849484; doi:10.1371/journal.pone.0263817)
Supplement: S1 Fig — (DOCX) [file pone.0263817.s001.docx]

**
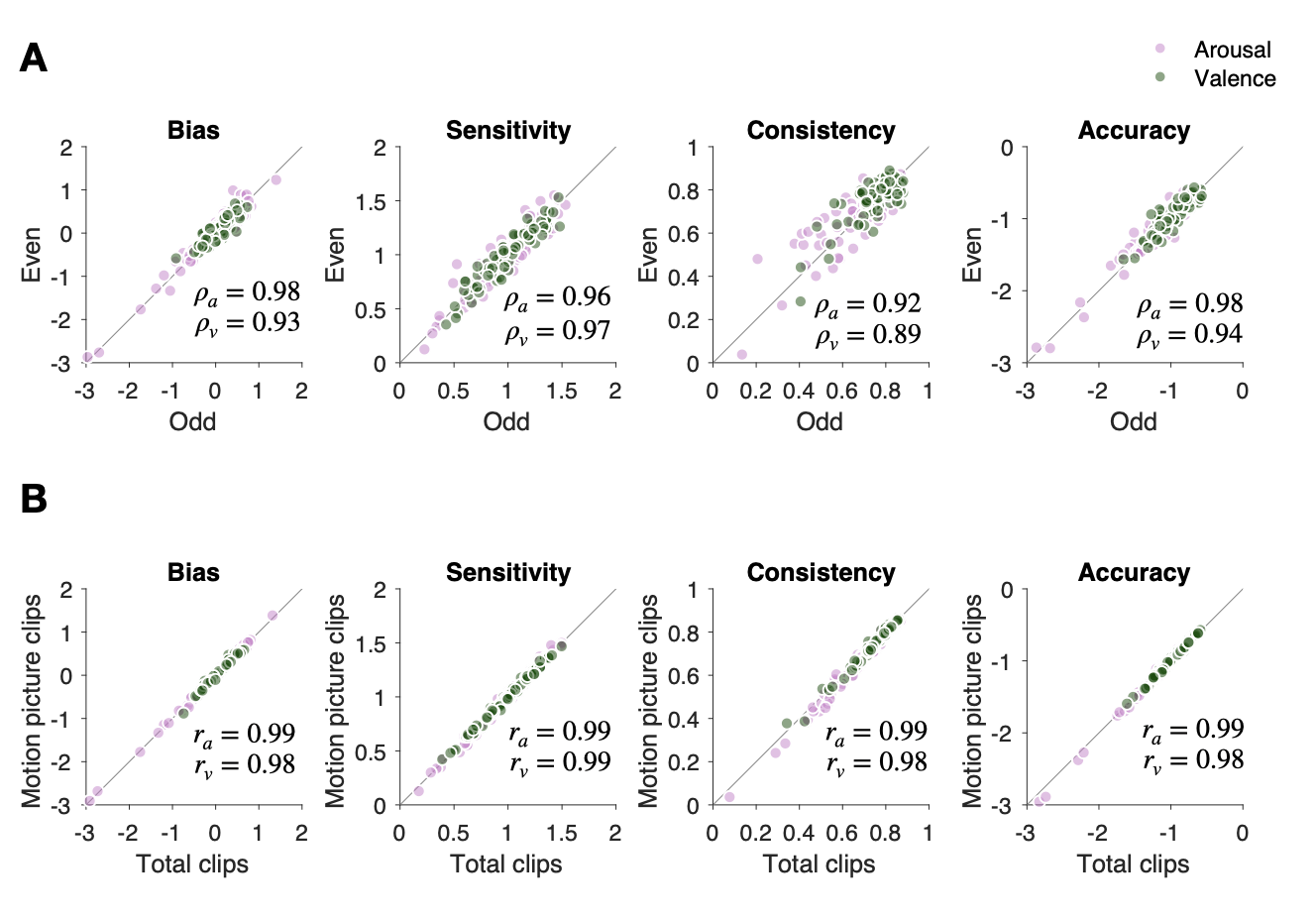
**

**S1 Fig.** **Reliability of emotion measures over visual narrative stimuli.**

(A) Split-half reliability. To evaluate how consistently visual narrative stimuli contribute to the reliable estimation of emotion measures, we carried out split-half reliability tests for all of the 8 emotion measures, $\left\{ \alpha_{a}^{i}, \beta_{a}^{i} \delta_{a}^{i}, {\sigma_{a}^{i}, \alpha}_{v}^{i}, \beta_{v}^{i}, \delta_{v}^{i}, \sigma_{v}^{i} \right\}$. For each type, two sets of emotion measures were separately estimated by calculating emotion rating responses to two separate halves of visual narrative stimuli on their respective normative (i.e., across-participant averaged) responses. Here, to match the representativeness between the halved sets, the visual narrative stimuli were first sorted by emotion ratings of relevance (i.e., ‘arousal’ (pink), ‘valence’ (green)) and then split into odd- and even-ranked sets. The split-half reliability was evaluated using the Spearman-Brown formula: $\rho=\frac{2r}{1+r}$ ,where $r$ is the correlation coefficient between the even and odd sets. For all of the 8 types ($\alpha_{a}^{i}, \alpha_{v}^{i}$ for the first panel; $\beta_{a}^{i}, \beta_{v}^{i}$ for the second panel; $\delta_{a}^{i}, \delta_{v}^{i}$for the third panel; $\sigma_{a}^{i}, \sigma_{v}^{i}$for the fourth panel), the emotion measures were consistent between the odd and even sets. (B) Reliability between the emotion measures acquired with the entire VN stimuli (144 video clips) and those acquired only with the VN stimuli excerpted from motion pictures (130 video clips). The 14 non-motion-picture clips were excerpted from music videos or commercials. The reliability was evaluated by computing the Pearson correlation coefficients between the two sets. The format and arrangement were identical to those used in A.
