# Supplementary material for: A robust multivariate structure of interindividual covariation between psychosocial characteristics and arousal responses to visual narratives
Source: PLoS One. 2022 Feb 16;17(2):e0263817. doi: 10.1371/journal.pone.0263817 (PMC8849484; doi:10.1371/journal.pone.0263817)
Supplement: S2 Fig — (DOCX) [file pone.0263817.s002.docx]

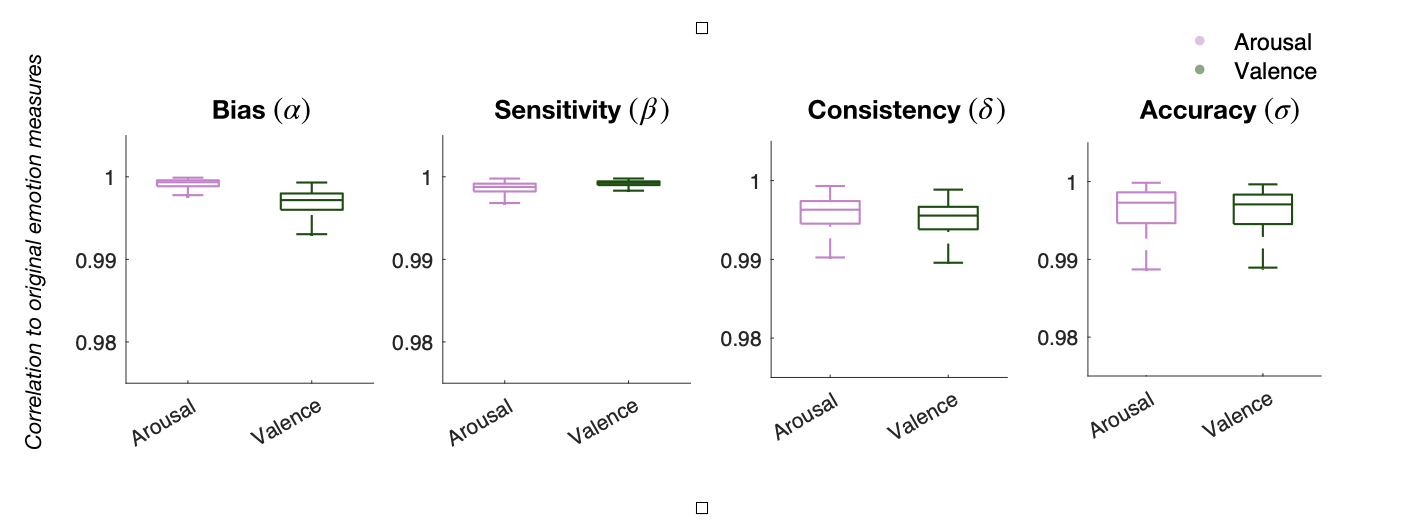


**S2 Fig.** **Invariance of emotion measures to different ways of defining normative emotion responses.**

To investigate to what extent emotion measures are affected by the different ways of defining the normative (across-participant averaged) responses, we repeated the calculation while varying the sub-population of participants that contributed to the normative responses. The size of the subgroup was fixed to 40. For each of the 8 emotion measure types, the calculation was repeatedly conducted on 2,000 different pairs of normative response sets. As a result, there were 8 sets of calculations. For each of these sets, we computed the across-participant correlations of the emotion-measure scores from these samples with the original emotion-measure scores and plotted the medians and ranges of correlations ($\alpha_{a}^{i}, \alpha_{v}^{i}$ for the first panel; $\beta_{a}^{i}, \beta_{v}^{i}$ for the second panel; $\delta_{a}^{i}, \delta_{v}^{i}$for the third panel; $\sigma_{a}^{i}, \sigma_{v}^{i}$for the fourth panel).
