# Supplementary material for: A robust multivariate structure of interindividual covariation between psychosocial characteristics and arousal responses to visual narratives
Source: PLoS One. 2022 Feb 16;17(2):e0263817. doi: 10.1371/journal.pone.0263817 (PMC8849484; doi:10.1371/journal.pone.0263817)
Supplement: S3 Fig — (DOCX) [file pone.0263817.s003.docx]

**
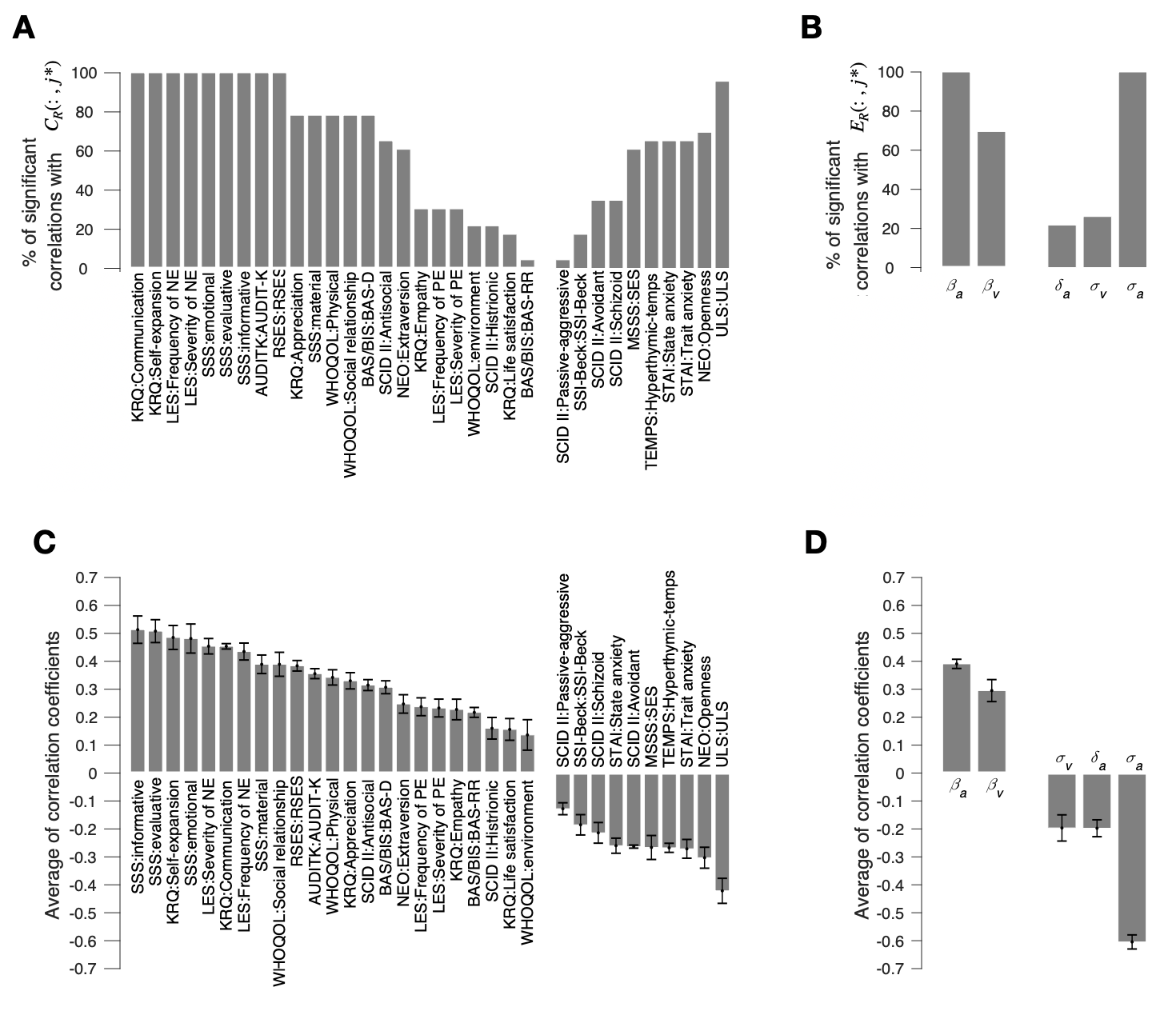
**

**S3 Fig.** **The detailed contributions of the psychological characteristics and emotion-response measures to the CCA mode**

(A, B) The proportion of CCAs in which given psychological-characteristics measures or emotion-response measures showed a significant correlation with their corresponding CCA variates ($C_{M1}$ or $E_{M1}$). There was a total of 23 different CCAs, which differed in the number of principal components that were used as input matrices. Only the measures that showed equal to or more than one significant correlation with the CCA variates are shown. (C,D) The across-variate averages of the correlations of the psychological characteristics or emotion-response measures with their corresponding CCA variates ($C_{M1}$ or $E_{M1}$). Error bars, 95% confidence interval. KRQ, Korean resilience quotient; LES, life experiences survey; SSS, Social Support Scale; AUDIT-K, Alcohol Use disorder identification test; RSES, Rosenberg self-esteem scale; WHOQOL, world health organization quality of Life; BAS/BAS, behavioral approach/inhibition system; SCID- II, structured clinical interview schedule for DSM-IV Axis-II disorder; NEO, revised NEO personality inventory; SSI-Beck, Beck scale for suicidal ideation; MSSS, MacArthur scale of subjective social status; TEMPS, temperament evaluation of Memphis, Pisa, Paris, and San Diego; STAI, state-trait Anxiety Inventory; ULS, UCLA Loneliness Scale.
