# Supplementary material for: A robust multivariate structure of interindividual covariation between psychosocial characteristics and arousal responses to visual narratives
Source: PLoS One. 2022 Feb 16;17(2):e0263817. doi: 10.1371/journal.pone.0263817 (PMC8849484; doi:10.1371/journal.pone.0263817)
Supplement: S4 Fig — (DOCX) [file pone.0263817.s004.docx]

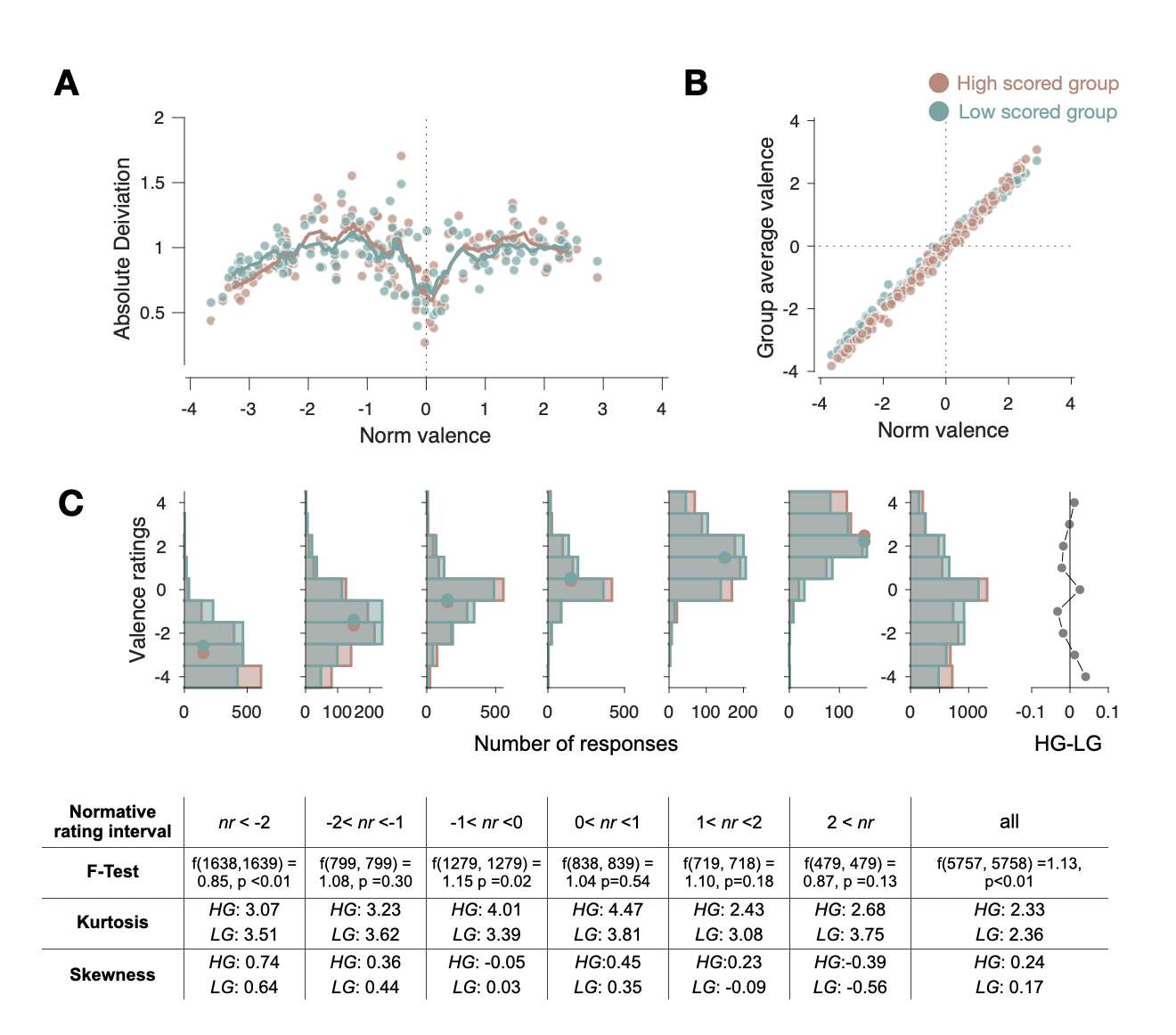


**S4 Fig. Distribution analysis on the valence responses.** (A) The averaged absolute deviations of valence responses from the normative responses plotted against the normative responses for the high (maroon dots) and low (teal dots) $E_{M1}$-score groups. The lines are the moving averages (window size, 10) of the averaged absolute deviations. (B) The averaged valence responses plotted against the normative responses for the high and low $E_{M1}$-score groups. (C) The comparison of the distributions of valence responses between the high and low $E_{M1}$-score groups. Top, the histograms of valence responses that are binned according to the normative response (six panels from left), the merged histograms of the entire valence responses (the second-rightmost panel), and the relative differences in proportion between the merged histograms (the rightmost panel, where 'HG' and 'LG' stand for the high and low $E_{M1}$-score groups, respectively). The histograms for the high and low $E_{M1}$-score groups are shown in maroon and teal, respectively. Bottom, the table summarizes the statistics of the histograms shown in the above. The columns’ locations are matched to the histograms that they describe. *nr* stands for the normative responses.
