# Supplementary material for: A robust multivariate structure of interindividual covariation between psychosocial characteristics and arousal responses to visual narratives
Source: PLoS One. 2022 Feb 16;17(2):e0263817. doi: 10.1371/journal.pone.0263817 (PMC8849484; doi:10.1371/journal.pone.0263817)
Supplement: S5 Fig — (DOCX) [file pone.0263817.s005.docx]

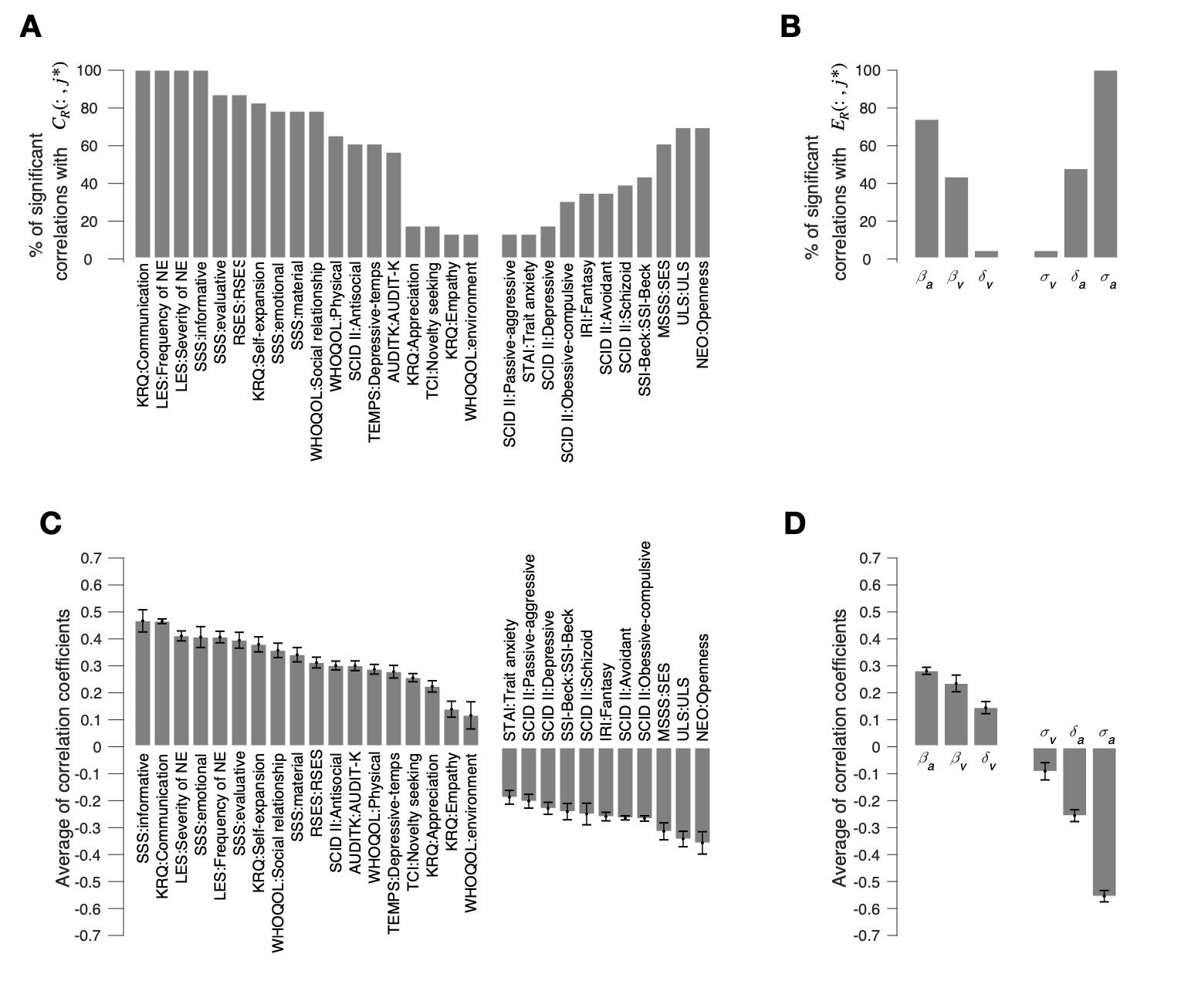


**S5 Fig.** **The results of the CCA analysis in which ‘extreme response style’ was regressed out.** The format is identical to that used in S4 Fig. KRQ, Korean resilience quotient; LES, life experiences survey; SSS, Social Support Scale; RSES, Rosenberg self-esteem scale; WHOQOL, world health organization quality of Life; SCID- II, structured clinical interview schedule for DSM-IV Axis-II disorder; TEMPS, temperament evaluation of Memphis, Pisa, Paris, and San Diego; AUDIT-K, Alcohol Use disorder identification test; TCI, temperament and character inventory; STAI, state-trait Anxiety Inventory; SSI-Beck, Beck scale for suicidal ideation; IRI, interpersonal reactivity index; MSSS, MacArthur scale of subjective social status; ULS, UCLA Loneliness Scale; NEO, revised NEO personality inventory.
