# Supplementary material for: A robust multivariate structure of interindividual covariation between psychosocial characteristics and arousal responses to visual narratives
Source: PLoS One. 2022 Feb 16;17(2):e0263817. doi: 10.1371/journal.pone.0263817 (PMC8849484; doi:10.1371/journal.pone.0263817)
Supplement: S1 Table — (DOCX) [file pone.0263817.s006.docx]

**S1 Table. Specifications of psychological-characteristic measures: Psychosocial factors category**

| **Questionnaires** | **Descriptions** | **Measures** |
| --- | --- | --- |
| KRQ  (Korean Resilience Quotient-53) | This measures the ability to cope with difficulties in life and to grow mentally by adapting to the environment. This is a modified version of the Resilience Quotient Test (RQT), which was developed by Reivich and Shatte [1], translated into Korean[2]. It consists of 9 sub-categories. A total of 53 items are rated on a 5-point scale from 1, “not at all true for me”, to 5, “very true for me”. | 1. *Appreciation*: degree of appreciation for everyday life 2. *Life satisfaction*: degree of satisfaction by focusing on what one can do well 3. *Self-positivity:* degree to which one believes things will get better 4. *Self-expansion:* degree to which one feels connected to others 5. *Empathy*: ability to read others’ thoughts and feelings 6. *Communication*: ability to establish and maintain relationships 7. *Causal analysis*: ability to see problems positively and find solutions accurately 8. *Impulse control*: ability to motivate and control oneself 9. *Emotion regulation*: ability to remain calm under pressure |
| SSS  (Social Support Scale) | This was developed by Park[3] to measure the degree of social support experienced by an individual. It consists of 4 sub-categories. Items are rated on a 5-point scale from 0 (not at all) to 4 (very much). | 1. *Emotional support:* experiencing respect, attention, affection, and trust 2. *Evaluative support*: receiving comments from others about one’s own behavior 3. *Informative support:* gaining information to solve personal problems 4. *Material support:* direct support when needed, such as money |
| RSES (Rosenberg Self-Esteem Scale) | This tool assesses individual global self-esteem, including both positive and negative feelings towards oneself. It consists of 10 items. The original version is scored on a 5-point scale, but the Korean version is scored on a 4-point scale from strongly disagree (1) to strongly agree (4). | It has no sub-categories.  *Example of an item:*  “On the whole, I am satisfied with myself.” |
| MSSS  (MacArthur Scale of Subjective Social Status) | This is a ten-point scale using a ladder model that measures subjective social status. The subject is asked to consider the ladder as a social structure and mark his/her social position.  This consists of two sub-categories. | 1. 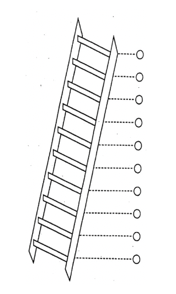*Traditional social status*: my position in social structure 2. *Community social status:* my position in my surroundings   Mark your position on a ladder. |
| LES  (Life Experiences Survey) | This is a self-report questionnaire that measures positive and negative experiences of the last year and their influence. It was developed by Sarason et al.[4], and was translated to and standardized for Korean[5]. Participants are asked to indicate whether they experienced 57 events, and, if so, to additionally rate those experiences on a 7-point scale from -3 (very bad) to 3 (very good). It takes about 10 minutes to complete. | 1. *Positive frequency:* frequency of experiencing positive events 2. *Positive severity*: sum of scores on positive events 3. *Negative frequency:* frequency of experiencing negative events 4. *Negative severity*: sum of scores on negative events |
| WHOQOL  (World Health Organization Quality of Life – BREF) | This is the Korean version of the WHOQOL-brief [6], which consists of 26 items, each rated on a 5-point scale (1, not at all; 2, a little; 3, a moderate amount; 4, very much; 5, an extreme amount). It has 3 sub-categories. | 1. *Physical health*: quality of life in physical health 2. *Social relationships*: quality of life in social relationship 3. *Environmental*: quality of life in environmental factors |
| IRI  (Interpersonal Reactivity Index) | This questionnaire measures empathy ability. It was developed by Davis[7] and translated into Korean[8]. It has 4 sub-scales and consists of 28 items, each scored on a 5-point scale (from “does not describe me well” to “describes me very well”). | 1. *Perspective taking*: tendency to put oneself in others’ positions 2. *Fantasy*: tendency to take the feelings and actions of virtual characters, such as characters in movies, as one’s own 3. *Empathic concern*: tendency to have feelings or interests toward others, such as warmth, sympathy, and compassion 4. *Personal distress*: tendency to feel uncomfortable and feel pain when seeing the unhappiness and suffering of others |
| ULS  (UCLA Loneliness Scale) | This is a self-report questionnaire that measures subjective loneliness and social isolation, and was developed by Russell and colleagues[9]. It consists of 20 items, each rated on a 4-point scale. | This has no sub-categories.  *Example of an item:*  “I feel left out.”  1. Never, 2. Rarely, 3. Sometimes, 4. Often |

References

1. Reivich K, Shatte A. The resilience factor: 7 keys to finding your inner strength and overcoming life's hurdles: Harmony; 2003.

2. Kim J. Resilience. Seoul, South Korea: Wisdom House.; 2011.

3. Park J-W. Study on the development of social support scale. Unpublished doctoral dissertation, Yonsei University, Seoul. 1985.

4. Sarason IG, Johnson JH, Siegel JM. Assessing the impact of life changes: Development of the Life Experiences Survey. Journal of Consulting and Clinical Psychology. 1978;46(5):932-46.

5. Lee Y. The Relations between attributional style, life events, event attribution, hopelessness and depression Seoul: Seoul National University; 1993.

6. Min SK, Lee CI, Kim KI, Suh SY, Kim DK. Development of Korean version of WHO quality of life scale abbreviated version (WHOQOL-BREF). Journal of Korean Neuropsychiatric Association. 2000;39(3):571-9.

7. Davis MH. A multidimensional approach to individual differences in empathy. JSAS Catalog of Selected Documents in Psychology. 1980.

8. Park S. Empathy, Empathic comprehension. Seoul: Woonmisa. 1994.

9. Russell D, Peplau LA, Cutrona CE. The revised UCLA Loneliness Scale: Concurrent and discriminant validity evidence. Journal of Personality and Social Psychology. 1980;39(3):472-80.
