# Supplementary material for: A robust multivariate structure of interindividual covariation between psychosocial characteristics and arousal responses to visual narratives
Source: PLoS One. 2022 Feb 16;17(2):e0263817. doi: 10.1371/journal.pone.0263817 (PMC8849484; doi:10.1371/journal.pone.0263817)
Supplement: S2 Table — (DOCX) [file pone.0263817.s007.docx]

**S2 Table. Specifications of psychological-characteristic measures: Clinical problems category**

| Questionnaires | Descriptions | Measures |
| --- | --- | --- |
| STAI  (State-Trait Anxiety Inventory) | This was developed by Spielberger and colleagues[1], and measures anxiety in normal adults. It is a self-report questionnaire consisting of 20 items that assess state anxiety and 20 items that measure trait anxiety. Items are rated on a 4-point scale from “almost never” to “almost always”. | 1. *State anxiety*: Undesirable short-lived feelings in specific situations 2. *Trait anxiety:* Undesirable long-lasing feelings in general situations |
| SCID-II  (Structured Clinical Interview Schedule for DSM-IV Axis-II Disorder) | This is used to diagnose psychological personality disorders[2]. It has been used to diagnose the Axis-II diseases in the DSM-lV system. Participants answer “yes” or “no” to a total of 119 items, each of which describes a certain experience or behavior under a certain situation. It has 12 sub-categories. | 1. *Avoidant*: A personality characterized by avoiding people since they are afraid of being rejected by others 2. *Dependent*: A personality characterized by being extremely dependent on others 3. *Obsessive-compulsive*: A personality characterized by being tied up with orderliness, rules, and the details of things 4. *Passive-aggressive*: A personality characterized by showing hostility or aggression in a passive way without direct expression 5. *Depressive*: A personality characterized by depressed mood, a lack of motivation, interest, and mental activity, irritability, loss of appetite, insomnia, constant sadness, and anxiety 6. *Paranoid*: A personality characterized by consistently having doubts of being betrayed by others or of being harmed by surroundings 7. *Schizotypal*: A personality characterized by social isolation, magical thinking, impaired consciousness, relationship delusions, and hallucinations 8. *Schizoid*: A personality characterized by no interest in social relationships (to be alone) 9. *Histrionic*: A personality characterized by extremely dramatic and exaggerated behavior and excitability to attract attention 10. *Narcissistic*: A personality characterized by exhibiting an extremely high self-importance or an excessive need for admiration 11. *Borderline*: A personality characterized by unstable fluctuations in emotion, behavior, and interpersonal relationships 12. *Antisocial*: A personality characterized by ignoring other people’s rights and feelings |
| SSI-Beck  (Beck Scale for Suicidal Ideation) | This was originally developed by Beck, Kovacs, and Weissman[3], and measures suicidal ideation during a clinician’s interview. Shin and colleagues[4] modified this to a self-report assessment (Cronbach’s alpha = 0.8), which was adopted in the present study. It consists of 19 items. | It has no sub-categories.  *Example of an item:*  “Do you have a desire to live?”   1. Strongly 2. Weakly 3. None |
| Audit-K  (Korean version of the Alcohol Use Disorder Identification Test) | This was developed by the World Health Organization (WHO) for early screening of diseases due to excessive alcohol drinking. We used the modified version of the Audit-K from Kim and colleagues[5]. It consists of 10 items. | It has no sub-categories.  *Example of an item:*  “How often do you drink an alcoholic beverage?”  0) never.  1) Monthly or less  2) 2–4 times a month  3) 2–3 times a week  4) 4 or more times a week |
| BAI  (Korean - Beck Anxiety Inventory) | This is a self-report questionnaire that measures anxiety symptoms. It consists of 21 items, and each item is rated on a 4-point scale from “not at all” to “severely”. | It has no sub-categories.  *Example of an item:*  “Feeling hot.” |
| BDI  (Korean - Beck Depression Inventory) | This is a self-report questionnaire that measures depression symptoms. It consists of 21 items, which include cognitive, emotional, motivational, and physical symptoms, and items are rated on a 4-point scale of 0 to 3. | It has no sub-categories.  *Example of an item:*  Sad.  0) I do not feel sad.  1) I feel sad.  2) I am sad all the time and I can’t snap out of it.  3) I am so sad and unhappy that I can’t stand it. |
| FTND  (Fagerström Test for Nicotine Dependence) | This is a shortened form of Fagerström Tolerance Questionnaire, which assesses the degree of physical dependence on nicotine. It was translated and standardized in Korea[6]. It consists of 6 items. | It has no sub-categories.  *Example of an item:*  “Do you smoke more frequently in the morning?”  1) Yes; 2) No |
| TEMPS-A  (Temperament Evaluation of Memphis, Pisa, Paris and San Diego auto-questionnaire version) | This tool measures 5 types of temperament. Participants are instructed to check whether each of the 110 items of temperament matches their own (yes/no). | 1. *Depressive*: e.g., “I am a sad, unhappy person.” 2. *Cyclothymic:* e.g., “My mood often changes for no reason.” 3. *Hyperthymic:* e.g., “I often get many great ideas.” 4. *Irritable:* e.g., “When angry, I snap at people.” 5. *Anxious:* e.g., “I am often fearful of someone in my family coming down with a serious disease.” |

References

1. Spielberger CD, Gorsuch R. State-trait anxiety inventory (form Y): Consulting Psychologists Press; 1983.

2. First MB, Benjamin LS, Gibbon M, Spitzer RL, Williams JB. Structured clinical interview for DSM-IV Axis II personality disorders: American Psychiatric Press; 1997.

3. Beck AT, Kovacs M, Weissman A. Assessment of suicidal intention: The Scale for Suicide Ideation. Journal of Consulting and Clinical Psychology. 1979;47(2):343-52.

4. Park KB, Shin MS. Perceived stress and suicidal ideation of high school students. Korean J Clin Psychol. 1991;10(1):298-314.

5. Kim C-G, Kim JS, Jung J-G, Kim S-S, Yoon S-J, Suh H-S. Reliability and Validity of Alcohol Use Disorder dentification Test-Korean Revised Version for Screening At-risk Drinking and Alcohol Use Disorders. Korean Journal of Family Medicine. 2014;35(1):2.

6. Park SM, Son KY, Lee YJ, Lee H-CS, Kang JH, Lee YJ, et al. A preliminary investigation of early smoking initiation and nicotine dependence in Korean adults. Drug and Alcohol Dependence. 2004;74(2):197-203.
