# Supplementary material for: A robust multivariate structure of interindividual covariation between psychosocial characteristics and arousal responses to visual narratives
Source: PLoS One. 2022 Feb 16;17(2):e0263817. doi: 10.1371/journal.pone.0263817 (PMC8849484; doi:10.1371/journal.pone.0263817)
Supplement: S3 Table — (DOCX) [file pone.0263817.s008.docx]

**S3 Table. Specifications of psychological-characteristic measures: Personality category**

| **Questionnaires** | **Descriptions** | **Measures** |
| --- | --- | --- |
| TCI  (Temperament and Character Inventory) | This is a self-report questionnaire that measures personality traits. It consists of 7 sub-scales and 240 items. | 1. *Novelty seeking:* Tendency to be stimulated by potential reward cues and new or mysterious stimuli 2. *Harm avoidance*: Tendency to be excessively worried about dangerous or repelled against disgusting stimuli 3. *Reward dependence*: Tendency to respond strongly to social compensation signals 4. *Persistence:* The tendency to sustain rewarded behaviors, even without any more rewards 5. *Self-directedness:* Ability to create situations to achieve his/her own goal and values 6. *Cooperativeness*: Ability to perceive oneself as a part of society 7. *Self-transcendence*: Ability to accept and identify the universe and nature, and feel unity with them |
| NEO  (Revised NEO-Personality Inventory) | This is an assessment of adult personality based on the Five-Factor model. We adopted a shortened form of the NEO-personality inventory that consists of 60 items, each rated on a 5-point scale from “very false for me” to “very true for me”. | 1. *Neuroticism*: Tendency to have impulsive and unstable emotions and difficulties in coping with stress 2. *Extraversion*: Tendency to interact with others and attract other people’s attention 3. *Openness to experience*: Tendency to exhibit a strong imagination, creativity, abundant emotion, having lots of ideas, and being artistic 4. *Agreeableness*: Tendency to help others by establishing intimate relationships and trusting others with warm feelings 5. *Conscientiousness*: Tendency to be confident, systematic, and deliberate in work processes, and to have a strong sense of responsibility |
| BAS/BIS  (Behavioral Approach/Inhibition System) | Several theories have suggested that the Behavioral Approach System (BAS) and the Behavioral Inhibition System (BIS) are crucial for personality; the former is to make a move toward something desired, whereas the latter is to regulate aversive motivation. A Korean version, modified by Kim and Kim[1], was used in our study to measure the sensitivity of each system. It consists of 20 items, each rated on a 4-point scale from “almost never” to “almost always”. | 1. *BAS Drive*: e.g., “I go out of my way to get things I want.” 2. *BAS Fun Seeking*: e.g., “I’m always willing to try something new if I think it will be fun.” 3. BAS Reward Responsiveness: e.g., “When I’m doing well at something, I love to keep at it.” 4. BIS: e.g., “I feel worried when I think I have done poorly at something important.” |

References

1. Kim K, Kim WS. Korean-BAS/bis scale. Korean J Health Psychol. 2001;6(2):19-37.
