# Supplementary material for: A robust multivariate structure of interindividual covariation between psychosocial characteristics and arousal responses to visual narratives
Source: PLoS One. 2022 Feb 16;17(2):e0263817. doi: 10.1371/journal.pone.0263817 (PMC8849484; doi:10.1371/journal.pone.0263817)
Supplement: S1 Appendix — (DOCX) [file pone.0263817.s010.docx]

**S1 Appendix. Power analysis and sample size justification**

**Methods**

To determine the number of participants that warrants sufficient degrees of specificity (1-$\alpha$, where $\alpha$ is the type I error rate), and of power (1-$\beta$, where $\beta$ is the type II error rate) as well, we proceeded as follows. First, following the convention, we initially set $\alpha$ to .05 for specificity. This initial $\alpha$ was corrected roughly to be .005 by Bonferroni correction to avoid the inflation of the type I error due to multiple comparisons intrinsic to our CCA analysis, which tests a total of 8 hypotheses. Second, following the convention, the power (sensitivity) was set to .8. Third, since the effect size for correlation comparison is defined as $Q$–the absolute difference between Fisher-z-transformed *r* values–and $Q$ of .3 is conventionally considered as a medium effect size [1], the target effect size was set to .3 in the unit of $Q$. Fourth, to derive the ‘null-hypothesis’ distribution of the correlations between the canonical variates paired in the first CCA mode ($r\left( C_{M1}, E_{M1} \right)$), we generated 10,000 bootstrap samples of data set from the 68 and 8 standardized normal distributions of the individual characteristic measures and the emotion measures, respectively, and carried out the PCA and CCA analyses using the same procedure described above. Then we transformed the 10,000 bootstrap samples $r\left( C_{M1}, E_{M1} \right)$ to Fisher z values, which provided us with the ‘null-hypothesis’ distribution of $z_{r(C_{M1}, E_{M1})}$. Fifth, we created the ‘alternative hypothesis’ distribution of $z_{r(C_{M1}, E_{M1})}$ by adding the target effect size ($Q$ = .3) to the ‘null-hypothesis’ distribution of $z_{r(C_{M1}, E_{M1})}$. Finally, we repeated the fourth and fifth steps as we vary the number of participants and determined the minimum number of participants that satisfies the pre-determined values of specificity (1-$\alpha$ =.995) and power (1-$\beta$ = .8). This analysis suggested 70 as the minimum number of participants (S1 Appendix. Fig1). Based on this result, we judged that eighty participants were sufficient for the multivariate analyses used in the current work.

**Results**


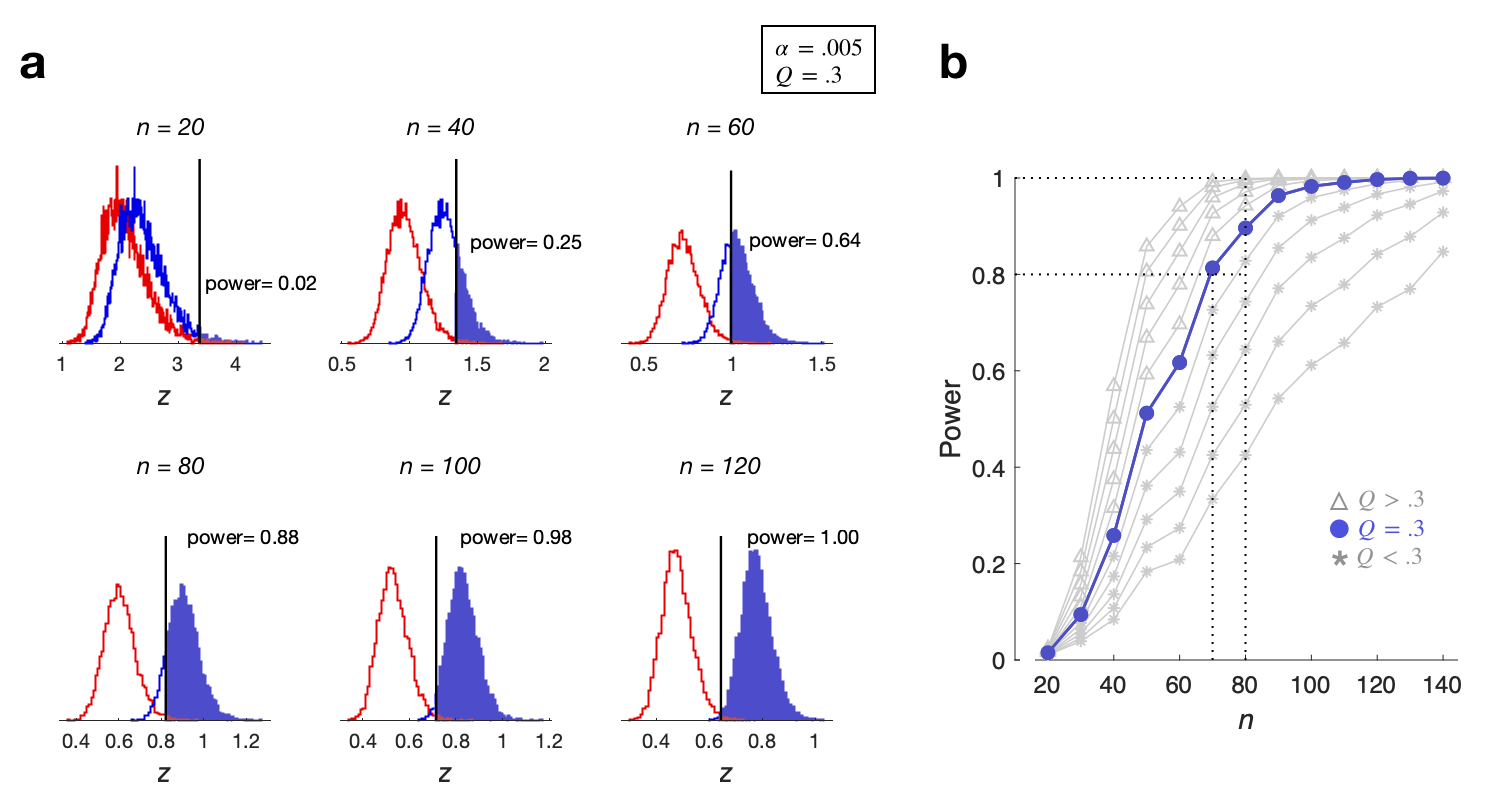


**Fig1.** **Results of simulation for sample size determination.**

(a) Example pairs of ‘null-hypothesis (red)’ and ‘alternative-hypothesis (blue)’ probability distributions of Fisher-z-transformed canonical correlation coefficient, with a pre-determined effect size (*Q* = .3). In each panel, the black vertical line demarcates the critical z value. The type I error ($\alpha$ = .005) corresponds to the area under the red curve for the z values that are greater than the critical value; The power (1- the type II error ($\beta$)) corresponds to the area under the blue curve for the z values that are greater than the critical value. As the number of participants (n) increases, the ‘null hypothesis’ and ‘alternative hypothesis’ probability distributions become increasingly apart from each other, which results in the increase in power. (b) Changes in power as a function of sample size under a fixed value of $\alpha$ (.005) and different effect sizes. Individual curves were acquired using different effect sizes, ranging from .2 to .4. With the pre-determined target effect size (*Q* = .3; blue curve), the power reached its pre-determined target level (.8) when the sample size was 70, as indicated by the left dotted vertical line. When the sample size was 80, which was the actual number of participants used for the data analysis, the expected effect size is greater than 3.8 and the expected power value is close to 1.

**Reference**

1. Cohen J. Statistical power analysis for the behavioral sciences. 2nd ed. Hillsdale, N.J.: Hillsdale, N.J. : L. Erlbaum Associates; 1988.
