# Supplementary material for: A robust multivariate structure of interindividual covariation between psychosocial characteristics and arousal responses to visual narratives
Source: PLoS One. 2022 Feb 16;17(2):e0263817. doi: 10.1371/journal.pone.0263817 (PMC8849484; doi:10.1371/journal.pone.0263817)
Supplement: S2 Appendix — (DOCX) [file pone.0263817.s011.docx]

**S2 Appendix. De-confounding the CCA for the overall tendency of making extreme reports**

**Methods**

We de-confounded the CCA for the across-participant variability in the overall (non-specific) tendency of choosing extreme reports [1] (e.g., going for 1 or 5, instead of 2 or 4, on the 5-point scale even when subjective feelings are rather moderate), as follows. First, we estimated this ‘extreme response style’ based on the patterns of reports shown in the NEO questionnaire. The NEO was chosen because it consists of the largest number of items (60 items) with 5-point scales and the individual items probe different aspects of personality traits so that the extremity of reports per se is unlikely to be associated with particular personality traits. Second, the extreme response style was quantified by calculating the percentage of endpoint responses (i.e., 1 or 5) over all the 60 items. Lastly, as was previously done for the socio-demographic variables, the extreme response style underwent a rank-based inverse normal transformation and then were regressed out from both $C_{P}$ and $E_{P}$prior to the CCA.

**Results**


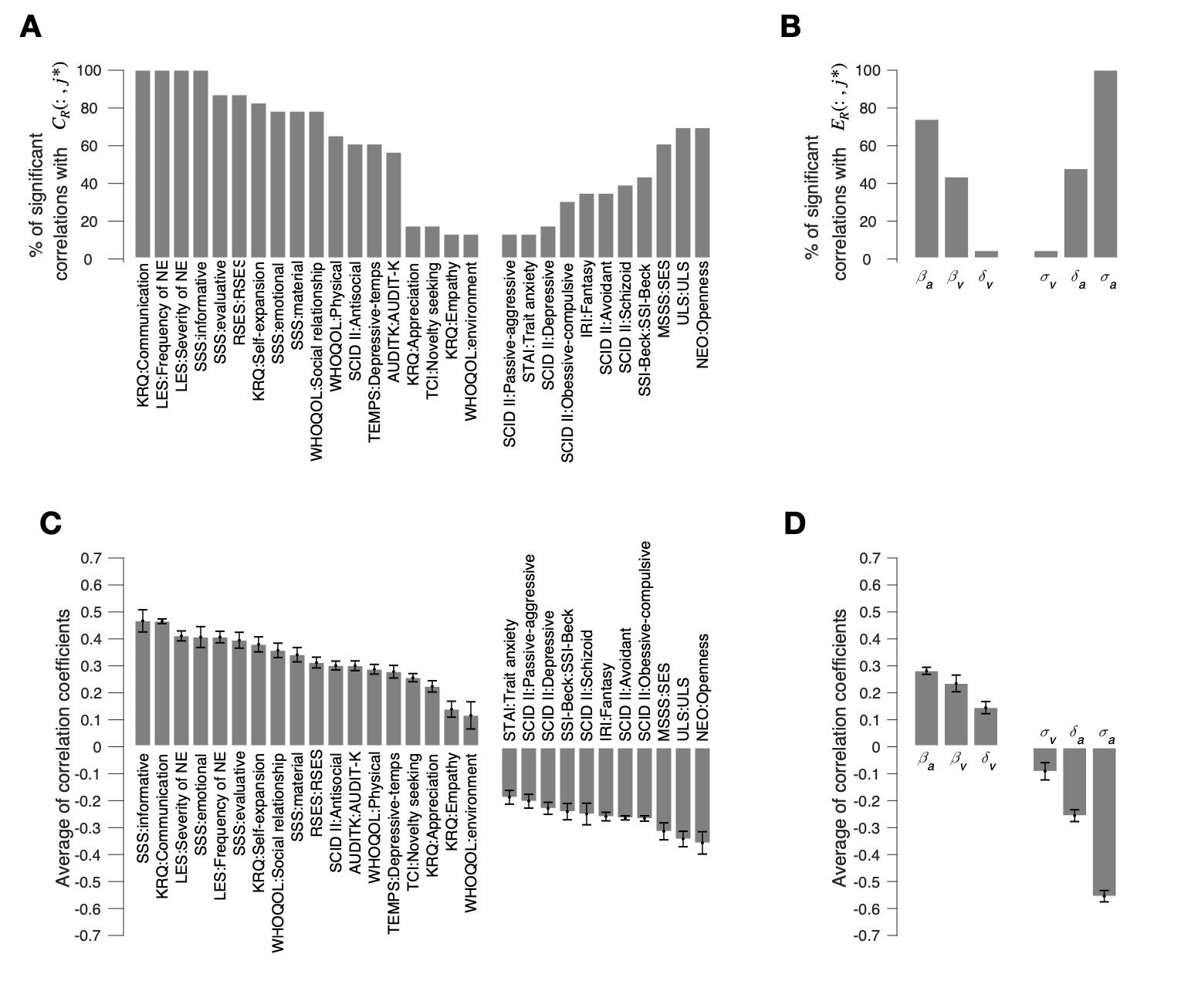


**Fig1.** **The results of the CCA analysis in which ‘extreme response style’ was regressed out.** The format is identical to that used in S4 Fig. KRQ, Korean resilience quotient; LES, life experiences survey; SSS, Social Support Scale; RSES, Rosenberg self-esteem scale; WHOQOL, world health organization quality of Life; SCID- II, structured clinical interview schedule for DSM-IV Axis-II disorder; TEMPS, temperament evaluation of Memphis, Pisa, Paris, and San Diego; AUDIT-K, Alcohol Use disorder identification test; TCI, temperament and character inventory; STAI, state-trait Anxiety Inventory; SSI-Beck, Beck scale for suicidal ideation; IRI, interpersonal reactivity index; MSSS, MacArthur scale of subjective social status; ULS, UCLA Loneliness Scale; NEO, revised NEO personality inventory.

**Reference**

1. Naemi BD, Beal DJ, Payne SC. Personality Predictors of Extreme Response Style. Journal of Personality. 2009;77(1):261-86. doi: 10.1111/j.1467-6494.2008.00545.x.
